# Human papillomavirus infection, cervical cancer and the less explored role of trace elements

**DOI:** 10.1007/s12011-022-03226-2

**Published:** 2022-04-25

**Authors:** Anne Boyina Sravani, Vivek Ghate, Shaila Lewis

**Affiliations:** Department of Pharmaceutics, Manipal College of Pharmaceutical Sciences, Manipal Academy of Higher Education (MAHE), Manipal, Karnataka 576104 India

**Keywords:** Cervical cancer, trace elements, chemotherapy, diagnosis, HPV

## Abstract

Cervical cancer is an aggressive type of cancer affecting women worldwide. Many affected individuals rely on smear tests for the diagnosis, surgery, chemotherapy, or radiation for their treatment. However, due to a broad set of undesired results and side-effects associated with the existing protocols, the search for better diagnostic and therapeutic interventions is a never-ending pursuit. In the purview, the bio-concentration of trace elements (copper, selenium, zinc, iron, arsenic, manganese, and cadmium) is seen to fluctuate during the occurrence of cervical cancer and its progression from pre-cancerous to metastatic nature. Thus, during the occurrence of cervical cancer, the detection of trace elements and their supplementation will prove to be highly advantageous in developing diagnostic tools and therapeutics, respectively. This review provides a detailed overview of cervical cancer, its encouragement by human papillomavirus infections, the mechanism of pathology, and resistance. Majorly, the review emphasizes the less explored role of trace elements, their contribution to the growth and inhibition of cervical cancer. Numerous clinical trials have been listed, thereby providing a comprehensive reference to the exploration of trace elements in the management of cervical cancer.

## Introduction

Non-communicable diseases account for a larger proportion of mortality rates reported in the world [1]. Among the various diseases, cancer is projected to be a major cause of mortality, resulting in 19.3 million cases (2020), split between 10.1million in men and 9.2million in women alike [2]. Though broadly termed as cancer, it represents diverse sets of diseases that can originate from and invade any organ in the body due to uncontrolled cell division [3, 4]. The cancers originating in lung and prostate tissues are the most common cancers reported clinically in males, with colon, stomach, and liver in close competition. However, in females, a high prevalence of breast, colon, lung, and cervical cancers is reported globally [5–7].

Cervical cancer accounted for 604,127 new cases (6.5% of all cases) and caused 341,831 deaths (7.7% of all deaths) in 2020 worldwide, marking its place as the fourth most fatal form of cancers in the female population [2]. Due to the ongoing demographic and epidemiological transitions, the global cancer burden is increasing rapidly, and it is expected to result in a significant share (> 4,74,000) of mortality among women by 2030 [8]. According to this global cancer burden, the incidence of cervical cancer is predominantly distributed between developed and less developed countries [9]. Additionally, the record of cervical cancer is declining in most countries owing to rigorous initiatives towards cancer screening and vaccination against human papillomavirus (HPV) infections, a primary factor of cervical cancer [10]. However, cervical cancer still haunts the population in less developed countries and those diagnosed between the ages of 15-44 years [11]. Women were highly susceptible to breast cancers, followed closely by colorectal and lung cancer for incidence and vice versa for mortality, making cervical cancer rank fourth for both incidence and mortality [1].

Cervical cancer is currently treated in numerous ways such as surgery [12], radiation exposure [13], chemotherapy [14], targeted therapy [15], and also destructive therapies like cryotherapy, chemical, loop electrosurgical excision procedure (LEEP), and photodynamic therapy [16–18]. Currently preferred treatment modalities using pharmaceutical interventions are often associated with severe side effects even though being extremely useful in the clinical setup. These include removal of uterus, cervix, lymph nodes, menstrual changes, swollen legs, hair loss, change in periods, increased infections, changes in the cervix, blood clots or bleeding [19–23].

Chemotherapy exhibits side effects that limit its application, requiring alternative approaches [24]. Trace elements such as copper (Cu), selenium (Se), manganese (Mn), iron (Fe), zinc (Zn) play an important role in cancer prevention [25–32]. These elements act as co-factors for antioxidant enzymes [33]. The essential trace elements have anti-cancer properties, and they exert their chemopreventive effect by aiding in the synthesis of antioxidant enzymes and removing the reactive oxygen species (ROS) [34]. Though indicated, a comprehensive report on the role of trace minerals in the pathophysiology of cancer is yet to be fully elucidated. For instance, Zn is a primary trace element responsible for homeostasis and is known to contribute to the etiology of cancer [35]. Within the cells, Zn majorly contributes to the stability of deoxy- and ribonucleic acid (DNA and RNA). Due to the higher dependency of RNA polymerases on Zn for their activity, these enzymes are known as metalloenzymes [36–38]. Supplementation of Zn promotes DNA synthesis, while its depletion inhibits DNA synthesis. As co-factors, Cu and Zn are involved in the biochemical reactions mediated by superoxide dismutase (SOD) [39, 40]. SOD is known for its role in protecting the tissues against free radical-induced damage and preventing cancer initiation [41].

Se functions as an anti-cancer agent by plausible mechanisms involving the immune system and development of cells [42–44]. The levels of Se and Zn were seen to drop lower than the healthy tissues in cervical intraepithelial neoplasia (CIN) and many other cancers [45, 46]. Co-administration of Se was suggested to suppress the ill effects of cisplatin-based chemotherapy on the kidneys and bone marrow, resulting in a higher therapeutic index of the drug [47]. Se plays an irreplaceable role as an antioxidant and in redox pathways, therefore prevents genetic damage [48]. As can promote the initiation and development of the tumor by epigenetic changes of critical oncogenes or tumor suppressors as gene promoters. Regulation of gene expression is linked to DNA methylation [49]. An increased level of As might lead to abnormal DNA methylation [50, 51].

## Methods

The statistical data on the prevalence and fatality of cervical cancer were collected from GLOBACAN 2018, International Agency for Research on Cancer (IARC), PubMed, Scopus, Web of Science databases, and the World Cancer Report 2020. The keywords used included: cervix, cervical cancers, HPV, human papillomavirus, progression, metastasis, resistance, diagnosis, screening, treatment, and management, combined with trace elements and nanotechnology. The search for clinical trials was carried out using PubMed and Scopus databases, including keywords such as advanced, recurrent, CIN, persistent and metastatic cervical cancer published (1984 to 2020) to identify clinical trials aimed at managing cervical cancer.

## Anatomy of the Cervix

**Fig. 1:**
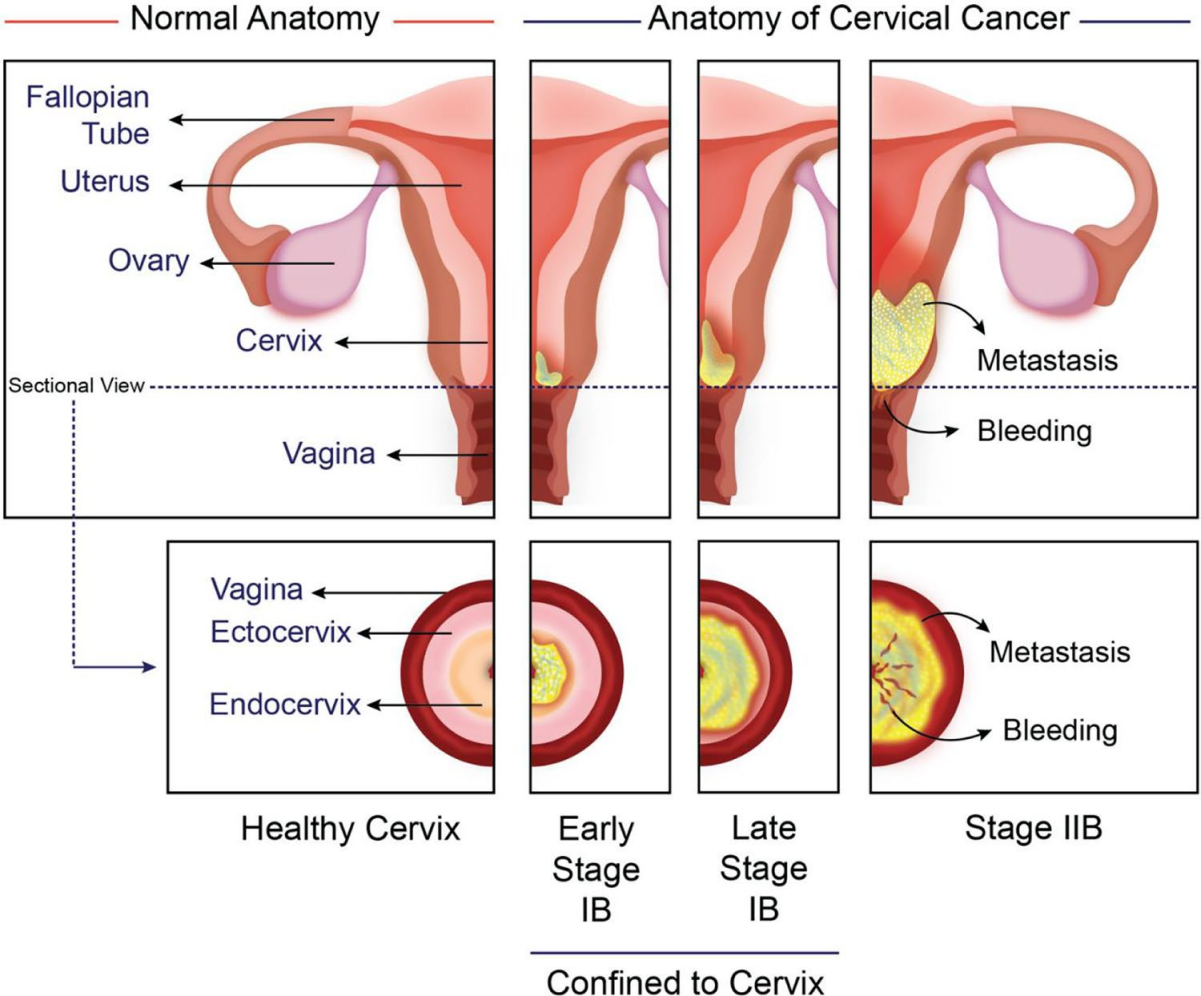
Anatomy of healthy cervix and the cancerous tissue showing the stages early and late stage IB, and stage IIB

The female reproductive system contains internal organs such as the uterus, vagina, fallopian tubes, and ovaries (Fig. 1). The vulva is another genital organ that is present externally. Pelvis holds the internal organs and is present between the bones of the hip in the lower abdomen [49]. Measuring about 3-4 cm and 1-3 cm in length and width, respectively, the cervix appears as a narrow portion of the uterus. It is a cylindrical to conical-shaped structure composed of stroma and epithelium. The major portion of the uterus is connected to the vagina by the cervix [52]. Cervix is formed with muscle and connective tissue and contains the ectocervix and endocervix, the two essential parts [53, 54].

The endocervix is the inner cervical part that lines the canal and finally ends with the uterus. There is a presence of leeway from the uterus to the vagina, which is called the endocervical canal. It is lined by the epithelial cells appearing tall and shaped like columns, also termed columnar cells. This columnar epithelium is mucus secretive with complex infoldings similar to the glands present on the cross section. The outer part of the cervix is called the ectocervix, which is round in shape and looks like a lip, is stitched into the vagina, and is lined, by squamous cells characterized by their flat and scaly appearance covered by non-keratinizing stratified squamous epithelium, which may be metaplastic or native. The ectocervix contains three layers, namely the basal layer, midzone layers, and superficial layer [49].

Both stratified non-keratinizing squamous and columnar epithelium cover the cervix. These two types of epithelium meet at the squamocolumnar junction [55, 56]. It is also known as the transformation zone as the column cells are continually being transformed into squamous cells. The same can be observed during pregnancy and puberty. The changes in precancerous conditions in the cervix as well as several cervical cancers originate from this transformation zone [57]. Part of the cervix consists of glands that make and release mucus. During the pregnancy and menstruation cycle, mucus becomes thick and stops entry of sperm into the uterus [58]. The thick mucus can also prevent the harmful bacteria from affecting the female's reproductive system (upper) and uterus.

### Diseases of cervix and cancer

The cervix is affected by many different conditions ranging from infections to cervical cancer [16, 59, 60]. The different disease conditions of the cervix are cervicitis, cervix poly, pelvic inflammatory disease (PID), cervical incompetence, Nabothian cysts, cervical myoma, cervical ectropion, endometriosis, HPV infection, cervical dysplasia, CIN, and cervical cancer [60–62].

**Fig 2:**
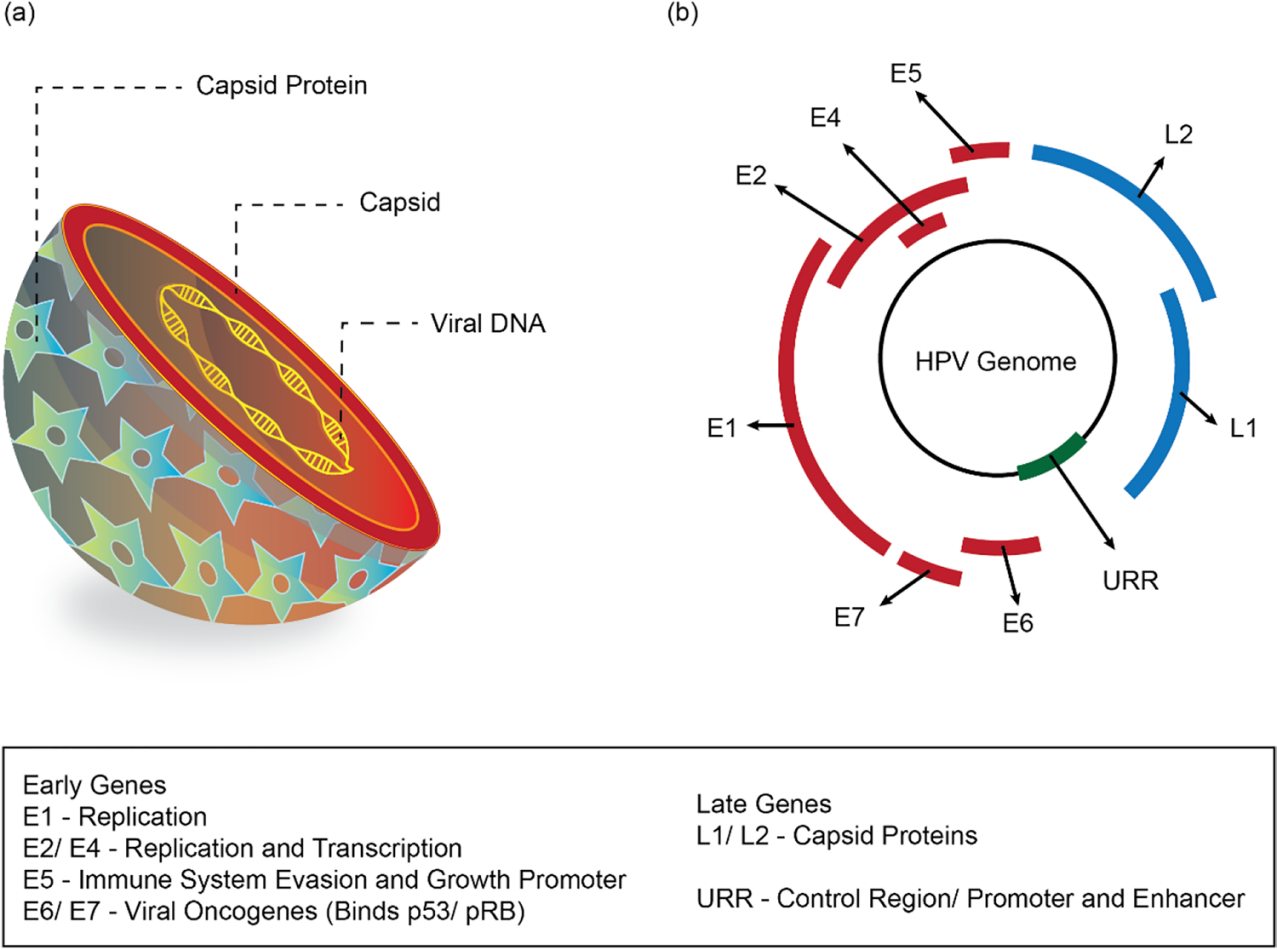
Human Papilloma Virus. Representation of the structure (**a**), and the genome organization (**b**)

HPV is a DNA virus and the main reason for cervical cancer [63]. The HPV-16 causes around 70% of cases of cervical cancer, and the remaining 30% of cases are caused by HPV-45, 18, and 31 [64]. The majority of HPV infections are caused by sexual intercourse when the cervical and vaginal epithelium are in contact with the virus [65, 66]. Though transient HPV infections are docile and can be easily cleared out of the body, some are known to cause dysplasia [67, 68]. Cervical cancer is classified into several types based on their origin within the cervix. Squamous cell carcinoma (SCC) originates in the ectocervix and accounts for ~90% of all cervical cancer incidences [19]. Cancer originating in the endocervix is termed adenocarcinoma [69]. Less common, adenosquamous carcinomas (or) mixed carcinomas may be seen overlaying features of both the SCC and adenocarcinoma [70]. Cells do not suddenly develop into cancer in the transformation zone. Instead, healthy cervical cells first undergo abnormal changes, called pre-cancerous changes [71–73]. For pre-cancers, there are several terms like CIN and squamous intraepithelial lesions (SIL) as depicted in the Fig. 2.

In CIN 1 (mild dysplasia or low-grade SIL), not much of the tissue seems abnormal and is considered as the less seriously affected cervical pre-cancer [74, 75]. More of the tissue appears abnormal in CIN2 or CIN3 (also referred to as moderate/severe dysplasia or high-grade dysplasia); among this, the most severe pre-cancer is high-grade SIL. Pap test is used to detect CIN before cervical cancer begins to develop [76–79].

Like all the other types of cancer, cervical cancer can be divided into several stages, accounting for its severity and metastasis [49, 80]. The cervical inner lining consists of abnormal cells referred to as *in situ* carcinoma (pre-cancer). The above abnormal cells will be developed into cancer and distribute to surrounding healthy tissues (Fig. 1). The cancer cells are only present in the cervix in Stage I (subdivided into IA and IB). Depending on the tumor size, IA(1-2) and IB(1-2) are the different stages of division. Stage II distributes over the cervix and is classified into IIA/ IIB based on the lower vagina or pelvic wall distance. Cancer progression to vaginal lower thirds and renal complications were observed in stage III of cancer. Likewise, based on cancer spread, IIIA and IIIB are the two more stages in stage III. The cancer location divides Stage IV into IVA and IVB. Spread of cancer to the rectum and bladder is observed in Stage IV. However, spread to bones, distant lymph nodes, lungs, and liver can be seen in stage IVB [81].

### Pathophysiology of cervical cancer:

Around 90% of cervical cancers are caused due to HPV [82]. Other risk factors include unsafe sex, poor immune system, multiple partners, chronic ingestion of oral contraceptives, age, race, genetics, low socioeconomic status, smoking, chlamydia trachomatous infection, micronutrient deficiency, and dietary deficiency in fruit and vegetables [83–86].

The structural features of HPVs are well studied to date [87, 88]. HPVs are capsids of icosahedral containing viral DNA with a diameter of about 55 nm. It contains a little non-enveloped DNA (double-stranded) of approximately 8 kb. The HPV genome contains three different parts that host six early genes and two late genes, and an extended control area (LCR) or non-coding region (NCR). The genes formed early are encoded for the expression in undifferentiated or immediately differentiated keratinocytes of six viral proteins which are non-structural (E1-7). The late region encodes the terminally differentiated keratinocytes to express structured viral capsid proteins (L1 and L2) [89, 90]. The structure of HPV and genome organization is shown in Fig. 2. The regulatory genes formed early are responsible for combating the replication mechanism of the host and encoding late genes for structural proteins [91].

**Fig. 3:**
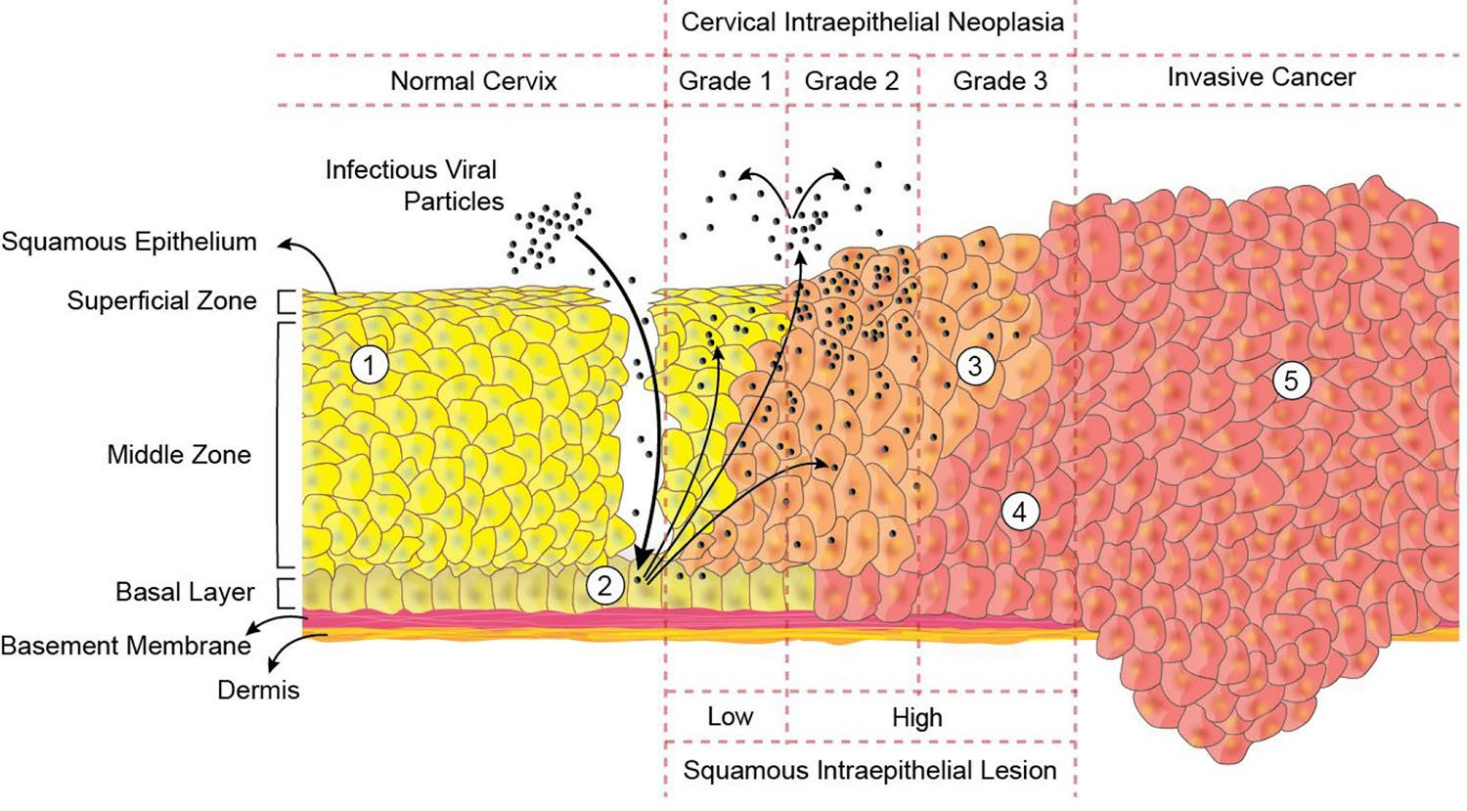
Histological considerations during cervical cancer graded as cervical intraepithelial neoplasia (CIN) and squamous intraepithelial lesion (SIL) depicting (1) the healthy squamous epithelium of the cervix, and observable changes during cancer including (2) the infection by human papillomavirus (HPV) with the episomal viral DNA in the nucleus of the cell, (3) cells expressing the early and late genes, (4) cells overexpressing the oncoproteins E6 and E7, and (5) the metastatic cancer with the complete integration of the viral DNA in the nucleus of the cells

During CIN, the cervical cancer cells mature, proliferate abnormally, and exhibit atypia.(Fig. 3) [74]. The grading of CIN is histologically done with regard to the area acquired by the dysplastic epithelial cells [92–94]. This epithelium consists of three regions, namely CIN 1, 2, and 3. CIN 1, also termed as a low-grade lesion, indicates atypical changes of the mild-level epithelium (basal 1/3^rd^). HPV is often present as viral cytopathic (koilocytoticatypia) [95, 96]. This relates to infection with HPV and is usually cleared by the immune response in a year, or it can take several years. CIN2 is known as a lesion of high grade, leading to moderate dysplasia, which in turn preserves the maturation of epithelium. CIN 3 can also be known as a lesion of high grade or dysplasia at a severe stage. The cytological classification of CIN is based on a three-tier scale indicates cellular changes which are seriously atypical, consisting of more than 60% of the epithelial thickness, and it includes *in situ* carcinoma. However, the new Bethesda diagnosis of cytological system classifies cervical cancer into high grade and low-grade squamous lesions [58].

### Mechanism involved in cervical cancer

**Fig. 4:**
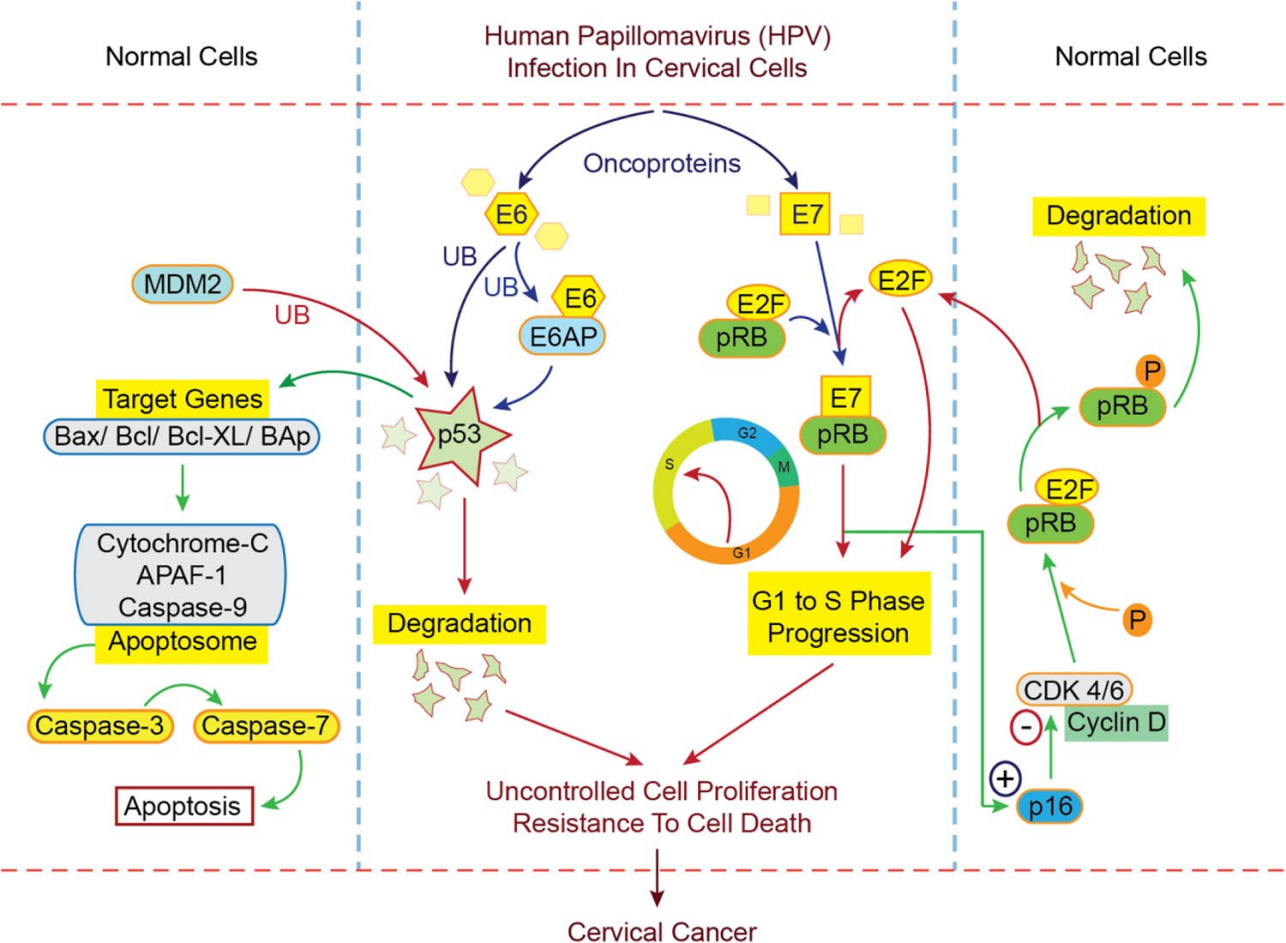
The mechanism of human papillomavirus (HPV) infection of the cervical cells, followed by the overexpression of the oncoproteins E6 and E7, leading to the derangement of the normal protective mechanisms of the cervical cells, resulting in the uncontrolled cell proliferation and resistance to cell death, termed as cervical cancer

The epithelium of metaplastic parabasal cells and basal cells is the source of origin for infection to HPV (Fig. 4). As the infection progresses, the viral genome is incorporated into the host cell. The manifestation of oncoproteins (E6/E7) following HPV infection results in the disturbance of the matured epithelial tissues [97–99]. The process further leads to the formation and development of abnormal epithelium. The lesions become fully formed thick epithelium from the early stages as the neoplastic process continues. As it proceeds, the cancer spreads to the surrounding healthy epithelium and tissues [66, 100]. The uncontrolled spreading may extend to the lymphatic system [101].

The prerequisite for cervical carcinoma is the continuous expression of E6/ E7 proteins [102]. These proteins can control the healthy normal cell cycle before integrating the virus, initiating the disruption of cells in the host [103]. Host cells respond to DNA damage, trying to repair the damaged structures including the epithelium. The cell division however continues to happen once the repairing process is completed within a damaged cell. If this repair is prohibited, apoptosis takes place [104, 105]. The inhibitors of the kinase (cyclin-dependent) and p53 degradation are the main reason for the disruption of the cell cycle by E6/E7, which in turn causes HPV-related carcinoma [104, 105]. With the degradation of p53, cells will continue to replicate continuously due to the induction of E6 oncoprotein [106, 107].

Further, p53 induces mitochondrial membrane permeabilization (outer) directly and stimulates the flow of proapoptotic factors [108]. The release of the mitochondrial cytochrome-c occurs as a response to the interaction of p53 with the Bax and Bak proteins [109–113]. Within the cytoplasm, an apoptosome is born with the adhesion of cytochrome-c to Apaf-1, activating the caspases (primarily caspase-9 followed by caspase-3 and caspase-7). These are indicators of the apoptotic pathway (intrinsic) [114, 115]. HPV will proceed with the response pathway eliciting damage, leading to replication and forming more number of HPV. The series of damage will favor the entry of HPV-DNA into the genome of the host. Hence, the proliferation of the cells will occur due to damage of pRb by E7 oncoprotein. HPV viral multiplication requires the cell to begin the S-phase [116]. This is executed by nullifying the pRb and free flow of the family of transcription factors (E2F), allowing the cell cycle progression from the G1 position [117–119].

Overexpression of E2F results in the cyclin D1 inhibition, kinase activity, and the kinase inhibitor 2A (CDKN2A, p16) gene product induction and overexpression [120–122]. With an association with Bax and Bak, E6 also interferes with the ongoing apoptotic processes [123, 124]. E6 is also known to regulate the proteins c-IAP2 and survivin leading to apoptosis resistance [64, 125]. E2F-1 inhibits Mdm2, resulting in the stabilization of p53 by stimulating P19ARF expression [126]. Several reports suggest an association of HR-HPV oncogenes with the transcription factors of the AP-1 group and as a stimulant for the mitogen-stimulated pathway of protein kinase (MAPKs) [127].

## Trace elements and cervical cancer

The alteration in the levels of trace elements is necessary for the pathogenesis of cervical cancer [128]. Trace elements, however, present in limited amounts in the circulatory system of human, play a significant role in several reactions of biochemical and enzymatic importance. These reactions when uniquely analyzed, are more important in causing several ailments and serious cancers [129, 130]. Trace elements are important for the normal development of the body, emphasizing the differentiation of several tissues [131]. In addition, trace elements are required for the survival of cancerous cells [132]. The biological role of trace elements, particularly Cu, Zn, Se, Fe, As, Cd, and Mn, influences occurrence, incidence, proliferation, and mitigation of tumors [133].

Zn is an essential trace element that is integral to many enzymes and transcription factors that regulate key cellular functions such as the response to oxidative stress, DNA replication, DNA damage repair, cell cycle progression and apoptosis. Zn deficiency effects the proper functioning of DNA and RNA polymerases and transcription factors [134–137]. Zinc deficiency also disrupts the function of both signaling molecules and proteins directly involved in DNA replication and repair. The mitogenic activity through pathways like AP-1, protein kinase, p53, and NFkB are modulated by Zn [138]. Limited availability of cellular Zn due to Zn deficiency could result in a loss of activity of these Zn dependent proteins involved in the maintenance of DNA integrity and may upregulate expression of the tumor suppressor protein p53, but impair the DNA binding abilities of p53, nuclear factor kB (NFkB) and AP-1 transcription factors [139]. This suggests that decrease in cellular Zn causes DNA damage and impairs DNA damage response mechanisms, resulting in a loss of DNA integrity and potential for increased cancer risk [140]. Zn in the body is usually associated with several macromolecules and metalloproteins formed from cancerous cells [38, 141]. Normal levels of Zn in neoplastic disease are often associated with low peripheral zinc bioavailability because zinc tends to bind to proteins with higher zinc binding affinity [142]. In addition to this, compounds containing Zn are used as photosensitizers for tumors, protective agents against radiation, antibacterial, antidiabetic (mimetics of insulin), and antineoplastic agents [143]. On the other hand, the levels of Cu are seen to increase in the serum due to malignant conditions, thus establishing a positive correlation between Cu levels and various grades of CIN [144]. As the stages of cancer progressed, the levels of Cu also increased, showing a linear progression.

Se is one of the key components of the anti-oxidant mechanism, seen in every cell; hence it possesses anti-cancer properties [145]. It was found that the low Se status resulted in the risk of cancer, whereas abundant Se resulted in protection against neoplasm [48, 146]. The medications fortified with Se have gained tremendous demand in the market. They tend to reduce the occurrence of side effects and increase immunity, overall leading to the suppression of cancer [147].

### Diagnosis of cervical cancer

#### Use of metals and trace elements

The time duration for change from pre-cancerous to infiltrative cervical carcinoma is generally ten years [148]. Cervical cancer screening is designed to identify the invasive form of cancer and aid in treating women with high-end cervical neoplasm (CIN2 and grade three SIL). Screening efficacy is measured by the reduction of cervical cancer occurrence and mortality achieved after screening [149]. Classical Papanicolaou smear test is a cost-effective method and has been extensively used in recent decades worldwide [77, 150]. Nonetheless, misdiagnosis happens regularly due to ~49% sensitivity and ~50.5% false-negative rate. The quality of the Thin Prep Cytological Test (TCT) smear is higher than the Papanicolaou smear, with a much faster exam speed and lower false-negative rate (~12.8%). However, the TCT requires a relatively expensive instrument and high cost of testing limits the outreach of the technique in developing countries [151, 152].

Many detection methods have recently been developed for HPV diagnosis, in which Hybrid Capture II (HCII) is one of the most widely used methods, with relatively higher sensitivity and specificity [153]. Nevertheless, the single positive findings of HPV cannot prove cervical cancer and should be followed by a specific diagnosis of cytology and histopathology. Vaginoscopy identifies and detects micro-lesions in the cervical and lower reproductive tract, providing a precise position for directing biopsy. However, the intracervical tube lesion cannot be observed by the vaginoscopy. Screening methods for cervical cancer can also differ based on the socioeconomic status of the areas [154]. Therefore, low-cost and highly efficient methods are essential in large-scale cervical cancer screening clinics.

##### Zinc and Copper

Zn a trace element has an essential role in nucleic acid, carbohydrates and protein synthesis [155]. Zn is used for the growth of the cell and in maintaining the integrity of the cell membrane. The cancerous cell may consume the Zn which is present in the circulation for tumor growth and maintain membrane integrity [156]. This might be the possible reason for the depletion of Zn levels in cancer patients [157]. It plays an anticarcinogenic role during the synthesis of DNA, transcription of RNA, immune system aspects, cell division, and growth [158, 159]. Zn is an integral part of bio-membranes besides its involvement in injuries related to peroxidation, stability, and membrane integrity control [160–163]. However, the role of Zn in carcinogenesis is linked to its function in the DNA and RNA polymerases, seen as inhibition of phosphodiesterases and promotion of membrane-bound adenyl cyclase [164]. Zn is also known to be a free-radical scavenger or antioxidant, and its deficiency may cause formation, transformation, initiation, and promotion of malignant cervical cancer [155].

As a co-factor of many redox enzymes, Cu will bind to nucleic acids and proteins, which may lead to proteins and lipids oxidation [165–167]. Cu ions also enhance the formation of harmful free radicals. It is essential for the proper functioning of metalloenzymes like cytochrome-C oxidase, α-amylase, carbonic anhydrase, superoxide dismutase, tyrosinase, dopamine hydroxylase, ceruloplasmin, ALA synthase, catalase, uricase, and ascorbic acid oxidase [144]. Elevated serum Cu levels are reported in various types of cancers [33, 168, 169]. Cu can directly interact with the bases of DNA at G-C sites. Thea addition of Cu to DNA in-vitro mediated more extensive DNA base damage inducing more mutations [155]. Cu may also elaborate other free radical species such as OH. Therefore, the inactivation of certain tumor suppressor genes can lead to the initiation and progression of carcinogenesis. The elevation of Cu levels may be due to Cu from tissue to serum [144, 157]. Cu plays an important role in the carcinogenic process that may also be linked with its ability to bind with some proteins and mediates the involvement of cellular proliferation via the activation of angiogenic growth factors [170]. The elevated levels of Cu resulted in the initiation of angiogenesis which increases the blood supply for tumor growth [171]. The deregulation of oxidative stress impairs cell DNA repair mechanisms due to the overproduction of ROS and is an important mechanism for cancer development. The proportion of serum Cu and Zn plays a major role in determining the presence of malignant gynecological tumor and the stage of cervical cancer [138, 172].

##### Selenium

Se contributes to important biological processes like prevention of DNA damage, energy metabolism in membrane integrity, antioxidant role, and redox regulation [157]. The selenoproteins occurring within the selenocysteine family, contribute to the essential enzymatic and structural functions [173]. Se exhibits antioxidative mechanisms and exerts its chemopreventive effect in several ways. These include defense against oxidative damage and ROS scavenging as well as improving synthesis of antioxidant glutathione peroxidase (GPx) [174]. The major physiological role of GPx is to maintain appropriate low levels of hydrogen peroxide within the cells and reduce potential the damage due to free radicals. It also provides a second line of defense against hydrogen peroxide that may create damage to membranes and DNA [175, 176].

##### Other trace elements

The significant levels of Fe in the body promote tumor development and are also associated with high mortality or neoplasm [172]. Fe is a vital trace element for normal cell functions, whose increased oxidative stress resulting in accelerated tissue and DNA damage [177]. Elevated serum Fe levels may increase the risk of lung, liver, colorectal, pancreas, skin, prostate, and cervical cancer [152, 178]. Moreover, the low Fe levels may have a role in the prevention of infection and cancer [179].

Fe has similar biochemical and chemical properties to that of Mn. The absorption of these two at the intestine is an example of an interaction between these trace elements [180]. Mn is an essential trace element required for several metalloenzymes as pyruvate decarboxylase and superoxide dismutase that are concerned with energy production and antioxidant defense system. Therefore, higher serum levels of Mn were found in people with lung cancer, malignant lymphoma, cervical cancer, colorectal cancer, and lower levels in esophageal cancer [172]. Ca is an essential element for many critical biological systems. Its deficiency is a risk factor in colorectal cancer [181], and Ca inhibits basal cell hyperplasia and dysplasia of the esophageal epithelium in regions with a significant risk for cancer in the esophagus [182].

Ni acts as a potential trace element and has capability of promoting tumor growth via some mechanisms such as induction of DNA aberration and deletion, inhibition of intercellular transmission mechanism, inhibition of the maintenance of nucleotide excision, oxidative deformation and methylation of DNA. Higher Ni levels are considered a potential risk factor leading to lung, colorectal, prostate cancer and nasopharyngeal carcinoma [183]. A higher concentration of Ni acts as a defensive factor for HPV infection, which is influenced by several factors [180]. The elevated serum levels of As may trigger high-grade lesions occurring within the cervical tissues due to HPV infection or cancer [133]. Cd exposure is a potential risk factor for the development of certain cancers [31, 184, 185]. The mechanism of carcinogenesis for Cd involves an increase of oxidative stress produced via reducing antioxidant proteins involved in antioxidant defense, depleting GSH and protein- bound sulfhydryl groups [186]. Cd ion can inactivate a series of enzymes and proteins containing metals through direct binding to their sites or creating disturbance in the enzyme topography, damaging cellular membranes [180].

### Treatment of cervical cancer

Several procedures available for treating cancer include radiation, chemotherapy, hormone therapy, surgery, targeted therapy, and immunotherapy [187–190]. Surgery can be preferred for the initial stages of cervical cancer [191, 192]. Advanced-stage CIN usually requires radiotherapy or chemotherapy [193, 194]. Chemotherapy is considered to be an effective approach for cervical cancer treatment. Radiation and surgery are the other supplements for the same. Current treatments have limited cancer effectiveness due to higher levels of tumor heterogeneity and drug resistance [195].

As discussed in pathophysiology of cervical cancer, E6 is a major player in the incidents of CIN [103]. Molecularly, E6-E6AP-p53 complex leads to the destruction of p53, a vital mediator of pro-cancer transforming protein. The binding sites of E6AP and p53 facilitates the interaction of anthracenyl-terpyridine Cu^2+^ complex with E6 [196]. The complex stimulated *in vitro* E6 aggregation in cultured cells. Both E6 and E6AP are necessary for a hijacking mechanism like ubiquitination and degradation of p53. The aggregation suppressed the E6 role, making it unable to hijack p53 and therefore increased p53 cellular level. In searching for a better therapeutic modality against cervical cancer, Cu2^+^ can be indicated as a new therapeutic option.

However, it is to be emphasized that not only trace elements, but certain metals such as ruthenium (Ru), can be used to manage cervical cancer [197, 198]. Ru liposomes (Ru-Lip) and pristine Ru were investigated for cytotoxicity in HeLa cells [199]. Results showed that Ru-Lip was more cytotoxic than non-encapsulated Ru. Ru-Lip significantly stimulated the generation of ROS, leading to the drop in the membrane potential of the mitochondria, and cytochrome C (Cyt C). For exhibiting apoptosis in HeLa cells, Cyt C is released from mitochondria into the cytoplasm, which in turn activates downstream proteins like caspase-3 and 9. In addition, Ru-Lip induced DNA damage and blockade in the S-phase of cell cycle, leads to apoptosis in HeLa cells.

In a study, core-shell nanoparticles comprising of Fe-carbide-glucose oxidase (Fe_5_C_2_-GOD, Fig. 7) magnetic nanoparticles as a core and MnO_2_ shell were synthesized. The core was functioned to increase the enzyme payload and the nanoshell for the protection of GOD from premature leakage before reaching the target tissue [200]. In the slight acidic microtumor environment, the nanoparticles were effectively transferred into tumor tissue utilizing magnetic targeting and continuous catalyst reactions, providing an effective strategy for treating tumors with enhanced tumor specificities and minimal side effects on healthy tissues. In another study, silver nanoparticles using *Nepetadeflersiana* plant extract showed concentration-dependent cytotoxicity. They increased the intracellular ROS damaging the mitochondrial membrane and modifying the cell cycle in HeLa cells [201].

Many studies reported in the literature mention that the fluctuation in the levels of trace elements in the body leads to cancer. Increase in the serum levels of Cu, Ca, Fe, Se and reduction in serum levels of Zn, Ni, Mn are the risk factors for cancer. NPs have demonstrated potential to deliver therapeutic moieties or contrast agents to the target site with minimal side effects to healthy tissue [25]. Hence, trace elements when loaded in nanocarriers and given as supplementation to the body make them more efficient to fight against cancer. These trace elements increase ROS generation leading to cellular and mitochondrial membrane damage, which eventually results in toxicity to cancer cells [202–204].

The physicochemical photoluminescent properties of chalcogenide semiconductors have been widely studied over the last few decades [205]. The partial decomposition and emission of toxic ions (Cd^2+^) by nanocrystals of Cd to cause fatal conditions to cancer cells is one among them. A specific way of preventing Cd-based nanocrystal toxicity is to make them well protected and biologically inert [206]. Another study with protein-coated CdS nanocrystals synthesized in an aqueous solution of bovine serum albumin (BSA) was favorable for the design and formulation of nanocrystals of uniform size. BSA acted as a stabilizer and a coating material, which showed sufficient cellular toxicity in HeLa cancer cell lines [207].

Ligand-stabilized heterogeneous Au-Cu^2^-x-Se nanocrystals were developed for application in photothermal therapy with a coating of water-soluble ligands [208]. Within the human cervical cells, the photothermal warming of these nanocrystals induced the cell abscission after 10 minutes of laser irradiation. ZnO-based nanoplatforms are increasingly being utilized in biomedical applications [209, 210]. Interestingly, studies have shown that the cytotoxicity of ZnO nanoparticles is not dependent on the levels of soluble Zn^2+^ ions in the extracellular component of the culture medium but due to the direct contact with the cells or uptake by the cancer cells [211]. The research field in chemistry with clinical, biology, and ecology applications can be achieved by fluorescent chemosensing for ions of metals [212].

Fluorescent chemosensors have a range of advantages over other sensors due to their real-time monitoring, ease of handling, and intrinsic sensitivity [213]. Cu and Zn ions are biochemically significant in aqueous media because of their essential role in biological systems [214]. In a study, new benzoyl hydrazine chemosensor R was developed, which was sensitive and selective towards Zn^2+^ and Cu^2+^, showed reversible fluorescence off-on responses in a water based medium. The sensor showed an excellent detection of ions, and cytotoxicity study showed that Cu (II) and Zn (II) complexes, had acquired an anti-cancer effect through the induction of phenotypic changes and membrane permeability improvement which were constant with the cell death induction due to apoptosis [143].

### Clinical Trials

Trace elements in cervical cancer are explored with an intention of providing early diagnosis and treatment options. Researchers conducted several clinical studies to correlate the link between trace elements and cervical cancer. Some of the studies are discussed here, presenting an excellent relation between low serum Zn/Se concentrations with invasive cervical squamous cell carcinoma, as shown in Table 1. Se and Zn can protect cell membrane from oxidative damage caused by lipid oxidation and avoids oxidative stress [218]. However, if the balance of these trace elements in the body is lost, they can lead to denaturation of proteins and oxidative nucleic acid damage, resulting in oxidative stress. Therefore, the changes in serum Zn/Se levels are associated with invasive cervical squamous cell carcinoma but the exact role is still unknown [131]. It was also found that the levels of serum Cu are significantly increased. These trials provided a landmark for the diagnosis of cervical cancers. Some other studies correlated the serum concentrations of Se and Ni with HPV infection to aid in the early diagnosis.

**Table 1:** Clinical studies on the use of trace elements for the diagnosis of cervical cancer

Study type/design of the study	Trial site	Samples and Method of Analysis	Name of trace element	No. of Subjects	Obtained Values of Trace Elements	Test/ P-value	Outcome	Reference
Analytical Cross-sectional study	Lagos University Teaching hospital (LUTH), Lagos, Nigeria.	Blood samples analyzed by Atomic absorption spectrophotometer.	Cu, Zn, and Se	50 diseased patients (Mean age: 43.1±7.5 years) and 100 control subjects (Mean age: 40.9±11.8 years)	Patients: Cu – 86.6±15.5 μg/ml Zn – 70.1±11.7 μg/ml Se – 101.3±7.7 μg/ml Control: Cu – 82.8±20.3 μg/ml Zn – 105.8±16.5 μg/ml Se – 120.9±18.3 μg/ml	t-test 0.099 0.003 0.026	The findings of this study indicated that the cervical carcinoma with invasions is caused due to low Se and Zn levels with increased Cu levels..	[128]
Random selection, Multivariant analysis	Tumor Hospital of Xinjiang medical university, study is performed in Uyghur women.	. Blood samples analyzed by Atomic absorption and inductively coupled plasma atomic emission spectroscopy for estimation of As and Se.	As, Cd, Fe, Ni, Cu, Zn, Mn, Se	833 women, comprising of 150 CIN≥2 and 683 CIN<2 (again in this 551 HPV positive and 282 HPV negative) with a median age of 39.62±9.58 years	HPV positive Ni- 0.1821mg/kg Zn- 105.9822μmol/L Fe- 7.3485mmol/L Cu- 19.8746μmol/L Mn- 0.4879μmol/L Cd- 0.03618μmol/L Se-≥0.02mg/kg(42.47%) As-≥0.02mg/kg(60.44%) HPV negative Ni- 0.121mg/kg Zn- 102.6875μmol/L Fe- 6.5872mmol/L Cu- 22.1089μmol/L Mn- 0.5263μmol/L Cd- 0.0258μmol/L Se-≥0.02mg/kg(58.51%) As-≥0.02mg/kg(48.23%) **CIN2+** Control group: Ni- 0.0942mg/kg Zn- 93.6937μmol/L Fe- 7.2894mmol/L Cu- 24.1875μmol/L Mn- 0.4984μmol/L Cd- 0.0418μmol/L As-≥0.02mg/kg(81.33%) Se-≥0.02mg/kg(15.33%) Case group: Ni- 0.1584mg/kg Zn- 105.5670μmol/L Fe- 6.9153mmol/L Cu- 21.8746μmol/L Mn- 0.5493μmol/L Cd- 0.0359μmol/L As-≥0.02mg/kg(50.07%) Se-≥0.02mg/kg(55.78%)	Chi-square <0.001 0.087 0.485 0.251 0.145 0.003 <0.001 0.001 (for both +ve and –ve) p-value <0.001 0.068 <0.001 0.328 0.134 0.071 <0.001 <0.001	The infection of HPV and CIN2 was due to Ni and Se in low levels and As in high levels. In contrast, levels of Cadmium, Zinc, Fe, Mn, and Cu did not impact cervical lesions and HPV infection.	[215]
Comparison of diagnostic and FH assay	Hospital of the University of South China	Tissue samples	Iron Protoporphyrin (FH) in uterus epithelia cells	574 Women comprising 340 women (normal cervical or benign lesion), 155 women (pre-cancer lesion), 79 women (early infiltrative cancer) with an average age - 34.78±7.52 years	79 out of 374 females were diagnosed with phase infiltrative cancer or infiltrative carcinoma (13.76%). The clinical diagnostic value of FH indicated higher than 80% of sensitivity and specificity in diagnosing cervical cancer and pre-cancerous lesion with about 18% misdiagnosis but only a 6.47% miss-diagnosis rate.	Chi-square	The principle theory involved in this study is the activation of the oxygen sensor and hypoxia sensor in tumor cells, the activity of ROS has altered the quantification of polarity within cells and released FH in the hydrophobic nucleus of cellular proteins into free FH. The FH assay has demonstrated high value in cervical cancer diagnosis,	[216]
Case-control study	Universitas Padjadjaran-Dr. Hasan Sadikin General Hospital, Bandung, Indonesia	Blood samples analyzed by Fluorimetry for estimation of Se and Spectrophotometry for estimation of GPx	Se and GPx	19 women patients with a mean age of 48.1±9.5 years, 20 healthy subjects with a mean age of 40±8.8 years	Se Patient- 67.24±15ng/ml Control- 77.05±12ng/ml GPx Patient- 128.18±38∆mmol NADPH/min/L Control- 148.9±23∆mmol NADPH/min/L	Paired t-test 0.03 0.04	The concentration of serum Se and activity of GPx is significantly lower in cervical cancer subjects. Results showed that the activity of Se and GPx would play an important role in carcinogenesis of cervical cancer.	[34]
Case-control study	Dr.V.M. Govt Medical College, Shree Chhatrapati Shivaji Maharaj General Hospital and Shree Siddheswar cancer hospital, Solapur, Maharashtra, India.	Blood samples	Zn, Cu	120 patients with age of 25-65 years, 30 healthy subjects with the same age	Higher MDA and nitric oxide levels, lower levels of RBC-SOD, lower levels of Vit-C, lower levels of zinc, higher levels of Cu and higher levels of Cu/Zn ratio	t-test <0.001	The generation of free radicles initiated due to the peroxidation of lipids which in turn due to the elevated levels of MDA causing mutation damage to the nuclear DNA. This is a significant reason for associating with SOD activity. Cervical cancer progression is due to the peroxidation of lipids.	[157]
Case-control study	Yonsei University Medical center, Seoul, Korea	Blood samples and analyzed by Atomic absorption spectrophotometer	Zn, Se, GPx, MDA (malondialdehyde)	28 Patients with CIN of age 33-73 years, 36 patients with invasive cervical cancer of age 35-74 years, and 44 controls of age 38-74 years	Se CIN- 5.82±0.62μg/dl Cacx- 7.33±0.81μg/dl Control- 10.25±0.85μg/dl Zn Control- 106.0±7.33μg/dl CIN- 76.5±4.24μg/dl Cacx- 78.2±5.87μg/dl MDA Control- 3.03±0.22nmol/l CIN- 6.06±0.53nmol/l GPx Control- 63.4±8.32nmol/NADH/min/mgpt CIN- 49.4±5.76nmol/NADH/min/mgpt Cacx- 50.4±4.86nmol/NADH/min/mgpt	Duncan test	The study showed alterations in oxidative stress biomarkers and antioxidant system during both the pre-cancerous and stages of invasion. Since the CIN indicates an early stage in neoplastic processes, the eventual impact of the malignant disease process on serum biomarkers can be mitigated, making it useful for risk markers evaluation.	[33]
Case-control study	Shanxi cancer hospital, Taiyuan.	Tissue and serum samples analyzed by Atomic absorption Spectrometry and Atomic fluorescence spectrometry	Zn, Ca, Cu, Fe, Mn, and Se	40 cases of cervical cancer, 30 cases of uterine myoma, 50 Healthy subjects with an age of 30-65 years.	Serum levels Zn, Se and Ca were significantly lower, Cu, Fe concentration and Cu/Zn ratio were significantly higher in patients with cervical cancer than healthy subjects	Paired t-test	In the cervical cancer group, the ratio of Cu/Zn and Cu concentrations were potentially in peak levels than in healthy people and myoma group subjects.	[172]
Case-control study	Hospital of Oncology of Mexican institute of social security, 21^st^-century Medical center, Mexico City.	Urine samples analyzed by Radiochemical neutron activation analysis	Se	82 women comprising of 8 control subjects, 19 initial stages of the disease, 35 intermediate stage, 20 advanced cervical, uterine cancer with the age of 24 – 60 years.	Se in the urine sample 3.9 – 6.4 ppb in healthy women, 3.1 – 6.4 ppb in initial stage (IA, IB), 3.0 – 28 ppb in intermediate stage (IIA, IIB), and 1.8 – 9 ppb in advanced stage (IIIA, IIIB)	t-test	This study showed that a normal urinary excretion of selenium tends to occur during the first or initial stage of cervical and uterine cancer. An increase of this excretion is seen in the intermediate stage, accompanied by a decline in the final stages of illness.	[217]
Case-control study	Dept of Obstetrics, Gynecology and Chemical pathology, Chinese University of Hong kong.	Blood samples analyzed by Atomic absorption spectrometry	Cu and Zn	25 Patients with cervical cancer, 16 with cervical dysplasia, and 19 minor with gynecological ailments	Controls Cu- 18.1±4.0μmol/l Zn- 13.8±2.2μmol/l Cu/Zn- 1.32±0.27 Invasive carcinoma Cu- 19.5±3.7μmol/l Zn- 12.6±1.7μmol/l Cu/Zn- 1.60±0.48 Cervical dysplasia Cu- 19.6±3.9μmol/l Zn- 13.2±2.2μmol/l Cu/Zn- 1.51±0.38	t-test	In this study, the plasma Zn is lower, and the plasma Cu / Zn ratio in the malignancy group is higher, the values of these elements are not significantly useful as a diagnostic method because of its decreased sensitivity when compared with other variable methods like cervical smears, colposcopy, and biopsy.	[155]

Some researchers worked on treatment, and many options were inundated in the twentieth century. Certain studies compared the Zn levels in patients with controls after pre and post-treatment with chemo irradiation, as shown in Table 2. Studies found a potential relationship between the values of mean serum Zn and the treatment outcome of patients. Some other researchers explored the role of Se in treatment as the normal urinary excretion of Se tends to occur during the first or initial stage of cervical cancer. The increase in the excretion of Zn is seen in the intermediate stage.

**Table 2:** Clinical studies on the use of trace elements in the treatment of cervical cancer

Study type/design of the study	Trial site	Samples and method of analysis	Name of trace element	No. of Subjects	Observed Values	Test and P-value	Outcome	Reference
Case-control study	Regional Institute of Medical sciences and hospital, Imphal, Manipur	Blood samples analyzed by Di-Br-PAESA method	Cu levels before and after the treatment (surgery, chemotherapy, radiotherapy or combined)	50 patients with a mean age of 57.78±17.66years comprising of 8 – Stage I, 13 – Stage II, 14 – Stage III, 15 – Stage IV and 30 Controls with a mean age of 57.74±12.12years	Control: 121.85±5.54μg/dl Patient: Before treatment 202.5±10.6μg/dl 215.5±9.63μg/dl 229.33±16.55μg/dl 237.5±15.85μg/dl After treatment 160.03μg/dl 165.85±10.6μg/dl 175.33±3.05μg/dl 180.62±11.55μg/dl	t-test	The results of this study concluded that the increased levels of serum copper in cervical cancer correlates with the cancer stages. This suggests that copper can be used as a parameter for cervical cancer screening, and can also be used as a valuable prognostic marker to monitor disease activity.	[144]
Case-control study	Dept of radiotherapy of a tertiary health care institute of central India	Blood samples and analyzed by Atomic absorption spectrophotometer	Zn levels in patients as compared with controls after pre and post-treatment with chemo-irradiation.	34 patients and 34 healthy controls with a mean age of 47.54 years	Pre-treatment Control – 0.34074ppm Patient – 0.31882ppm Post-treatment Group1- 0.34179ppm (Complete response CR) Group 2 – 0.32290ppm (Partial response PR/No response NR)	Unpaired t-test 0.0775 0.0463	The mean Zn serum value in patients with cervical cancer who received CTRT (Concurrent Chemoradiation Therapy) and achieved a complete response (CR) was significantly higher than that in patients with partial response (PR) / no response (NR). It points to the potential relation between mean serum zinc values and the patient's treatment outcome. Furthermore, they found no substantial difference between controls and patients in the serum Zn levels.	[138]
Case-control study	Sri Venkateswara Medical College, Tirupathi, India.	Blood samples analyzed by Atomic absorption spectrophotometric method	Selenium levels before and after treatment with chemotherapy and radiotherapies	104 Cervical cancer patients comprising of 54 treated with chemotherapy (age: 46.13±4 years) and 50 treated with radiotherapy, and 50 Controls (age: 30-73 years)	Before treatment Control: 13.83±0.21μg/dl Patients: 7.32±0.59μg/dl After treatment Chemotherapy: 11.16±0.32μg/dl Radiotherapy: 8.90±1.23μg/dl	t-test and ANOVA	The findings revealed that chemotherapy, but not radiotherapy, had increased the levels of trace elements and antioxidant activity in the blood serum of patients with cervical cancer. Increased Se in the serum of patients with cancer induces increased production of Se-dependent antioxidant mechanisms like GPx.	[145]
Randomized multicenter study	Freiburg, Germany	Whole-blood samples	Selenium supplementation for reducing the side effects of patients treated with radiotherapy (RT) for cervical and uterine cancer	80 Patients with a mean age of 64.3±10.1 years comprising of 70 with uterine cancer, 11 with cervical cancer -grouped as 39 selenium group (SG), 42 the control group (CG)	Before RT Se supplementation – 65.3μg/dl Without Se – 63.2μg/dl 50% RT Se supplementation – 93.2μg/dl Without Se – 67.3μg/dl End of RT Se supplementation – 90.9μg/dl Without Se – 61.4μg/dl 6 weeks after RT Se supplementation – 73.2μg/dl Without Se – 69.0μg/dl	t-test and Mann-Whitney U-test 0.49 <0.001 <0.001 0.32	During RT, selenium supplementation is effective in improving blood serum status in patients with Se-deficient cervical and uterine cancer and reduced the number of episodes and frequency of RT-induced diarrhea.	[173]
Case-control study	Institute of Gynaecology and Obstetrics, Ancona University, Italy	Blood samples analyzed by Atomic absorption spectroscopy	Zn Supplementation	22 patients with locally advanced squamous cervical carcinoma, and 12 Healthy controls For both groups age is ≤60 years	Active thymulin (ZnFTS) (log^-2^) Controls: 3.0±0.3 Patients: 1.5±0.5 Total thymulin (ZnFTS + FTS) (log^-2^) Controls: 4.5±0.3 Patients: 4.5±0.3 Zinc Controls: 112.4±16.1μg/dl^-1^ Patients: 100.7±9.3 μg/dl^-1^ α2-Macroglobulin Controls: 178.8±17.2 μg/dl^-1^ Patients: 260.0±72.8 μg/dl^-1^	Student's t-test and ANOVA (one-way)	Active thymulin (Zn-FTS) was reduced in patients affected by locally advanced cervical carcinoma, whereas total thymulin level (active thymulin Zn-FTS + inactive thymulin FTS) was in the normal range. *In vitro* addition of Zn to plasma samples containing (FTS), revealing the total amount of thymulin (active+inactive) in the circulation. It was concluded that the ratio of total thymulin to active thymulin is the thymulin fraction that is saturable by Zn ions and represents a useful marker of true Zn deficiency and, consequently, of peripheral Zn which may be low despite plasma Zn levels in the normal range.	[219]
Case-control study	Radiotherapy Clinic at Pt.B.D.Sharma PGIMS, Rohtak, Haryana, India.	Blood samples and method analyzed Atomic absorption spectrophotometer	Se	25 patients with mean age 50.3 years and 20 healthy subjects with the same mean age	Se Control: 120.57±13.45ppb/ml Patient: 97.4±16.0ppb/ml	t-test	The findings of the present study suggested that low Se serum concentrations in patients with uterine cervical carcinoma might be a contributing factor in cervical cancer development. Se supplementation can play an essential role in carcinogenic chemoprevention.	[220]

## Use of Metallic Nanoparticles:

Nanotechnology plays a vital role in the accurate diagnosis, early detection, and treatment of malignancies [221, 222].

**Fig. 5:**
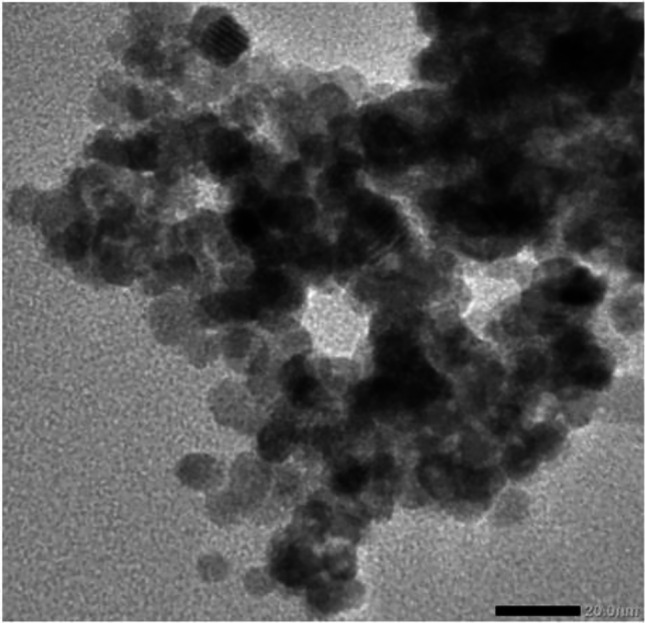
Transmission electron microscopic image of ZnO quantum dots. Reproduced under CC BY 4.0 from [232], Frontiers in Pharmacology

Quantum dots (QDs) are a cluster of autofluorescent semiconductor nanoparticles and have a promising outlook in various biomedical fields. They exhibit fluorescent properties because of their ability to absorb photons, which results in the formation of an electron-hole pair [223]. QDs, also known as an artificial atom, consist of high discrete electronic energy in a molecule or an atom [224, 225]. The photoluminescence of QDs emitting in the visible region can be modified by changing their size and composition [226]. Changing the size of QDs varies the emitted color. QDs can be made from metals or semiconductor material like Ni, Zn, Se, S, Cd [227]. Capping the core nanocrystals with ZnO has been shown to increase stability and performance, producing the QDs with improved luminescence and high photochemical stability [228]. CdZnS/ZnS-based blue QDs were synthesized in a study, presenting an excitation and emission at 405 nm and 450 nm, respectively, employed for cellular imaging. Capping of ZnS has been shown to increase stability and performance, producing the QDs with improved luminescence and high photochemical stability [228]. The resulting QDs were preferentially taken up by the cancer cells permitting their visualization by confocal microscopy. The results demonstrated QDs to be a stable alternative to other probes for imaging, cell sorting, and targeting applications [229]. The QDs are effectively used in revealing cancer invasion, focusing on the tumor environment, diagnosis, tumor imaging and treatment of cancer and are less toxic. The ZnS/ZnO involved in increased oxidative stress as well as inducing apoptosis [223, 230, 231]. In another study, the quantity of ROS was increased with ZnO QDs (Fig. 5) and inhibited the mitochondrial membrane potential in a correlation with the dose. Moreover, the ZnO QDs were shown to increase the early and late-stage apoptosis HeLa cells [223].

A different type of trace element-based QDs, namely AlSe/ZnSe QDs, exhibited profound biocompatibility in healthy tissues and uptake in cancerous cells confirmed by cell viability assays. Hence, these QDs can be added to photosensitizer to improve cancer therapy and serve as a biological imaging probe [233]. An electrochemiluminescence (ECL) system utilizing Zn-doped MoS_2_ QDs/ reductive Cu(I) particles showed excellent ECL due to hydrogen peroxide adsorption. Additionally, the reductive Cu(I) particles enhanced the ECL by catalyzing the co-reactant hydrogen peroxide, applicable for the biosensing of HPV 16 DNA [234]. In another study, CdSe quantum dots showed inhibitory effect on Rho-associated kinase (ROCK) activity in cervical carcinoma HeLa cells associated with the attenuation of the ROCK-c-Myc signaling. They demonstrated QD-mediated ROCK inhibition significantly and reduced c-Myc protein stability due to reduced phosphorylation as well as its activity in transcribing target genes (e.g. HSPC111). As a result of the reduced ability of c-Myc to drive cell proliferation, QD therapy significantly restrained HeLa cell growth by inducing cell cycle arrest at G1. Furthermore, since HSPC111, one of the c-Myc targets, is involved in controlling cell growth via ribosomal biogenesis and assembly, downregulation of HSPC111 could contribute to decreased proliferation in HeLa cells following QD treatment [235].

QDs and fluorescent semiconducting polymer dots (Pdots) also showed attractive attention as theranostic agents. They have excellent biocompatibility and remarkable optical properties like extraordinary photostability and a high quantum yield [236–240]. Super-resolution cell imaging, particle tracking in single, and cell labeling are the areas where Pdots are applied. In a study, octaarginine peptides (R8)-mediated cellular uptake and transportation of Pdots were realized by coating Pdots with synthetic R8 in live HeLa cancer cell lines. The majority of R8-Pdots entered the cells immediately when compared with unmodified Pdots. Also, the study showed increased autophagy in HeLa cells, implying their significance for direct regulation of cellular homeostasis besides functions as carriers of therapeutic agents and imaging probes [241].

Fermi's golden rule expresses a molecule's radiative rate. This rate is directly proportional to the medium density which was surrounding the molecule or atom [232, 242, 243]. The emission energy can be controlled by inserting a molecule in a photonic crystal. The emitted light directions and rate controls are also possible [244, 245]. Many photonic band-gap materials can be formed by conjugation with biological molecules, making them an attractive product for biomedical applications [246]. Surrounding environments of metal nanoparticles, the electromagnetic can be potentially enhanced, providing detection with the help of novel mechanisms. It was proved that the metallic nanocrystals might be used for sensitive and specific detection of nucleic acids by surface modification of gold nanocrystals. The metal nanoparticles will aggregate under excess ionic strength. This is one of the practical difficulties when working with nanoparticles. Oligonucleotide addition will avoid this difficulty of aggregation of metallic nanoparticles [247].

**Fig. 6:**
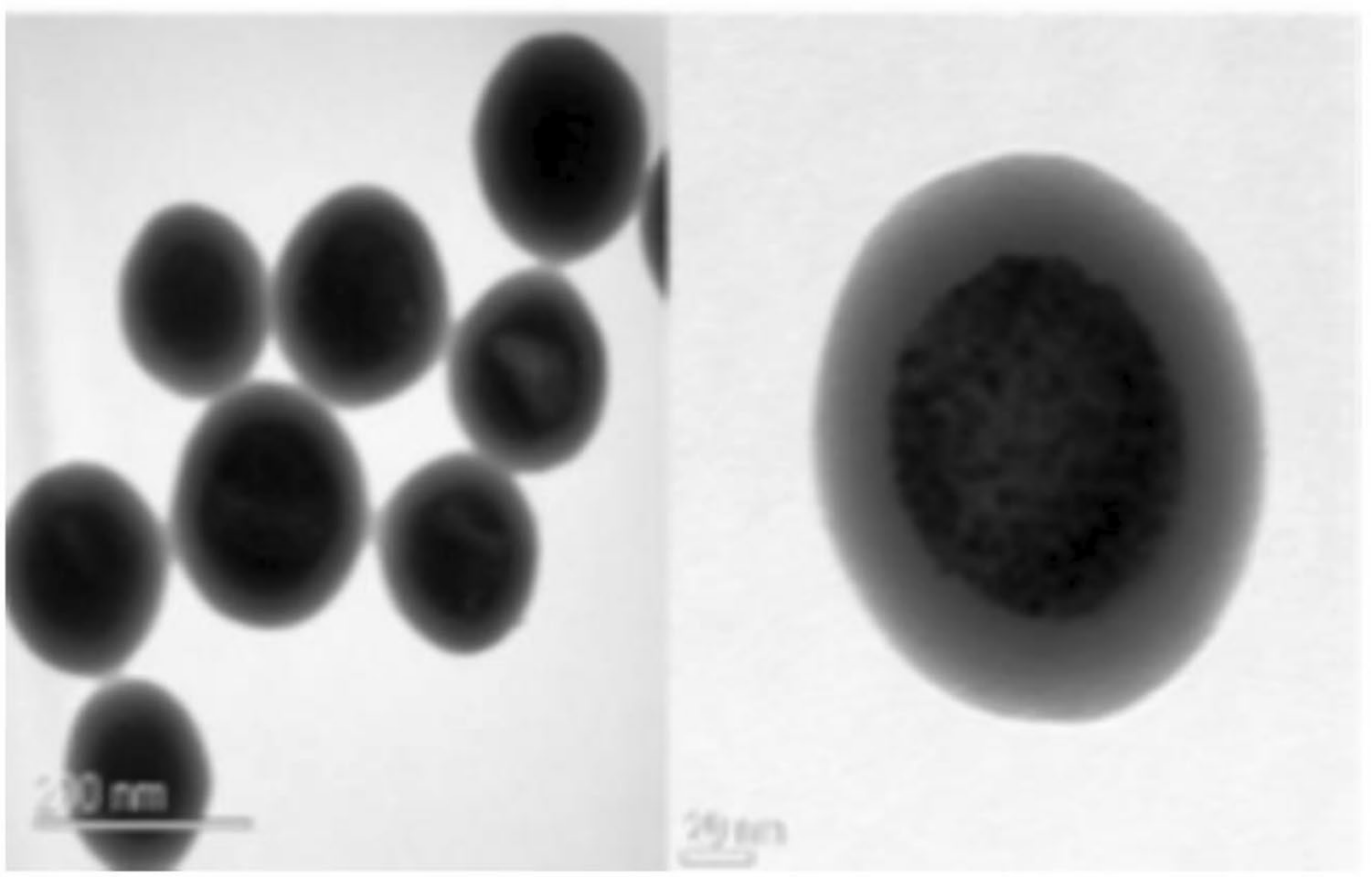
Transmission electron microscopic images of fluorescent gold-nanocrystals-silica hybrid (FLASH) nanocomposites. Scale bar—50 nm. Reproduced from [251] with permission from American Chemical Society (ACS)

Magnetic nanocrystals play a vital role in systems for separation and artificial detection biologically [248–250]. Fluorescent gold-nanocrystals-silica hybrid nanocomposite (FLASH, Fig. 6) prepared by the co-condensation method exhibited photodynamic activity against HeLa cells, making them a source for cancer therapy and bio-imaging [251].

**Fig. 7:**
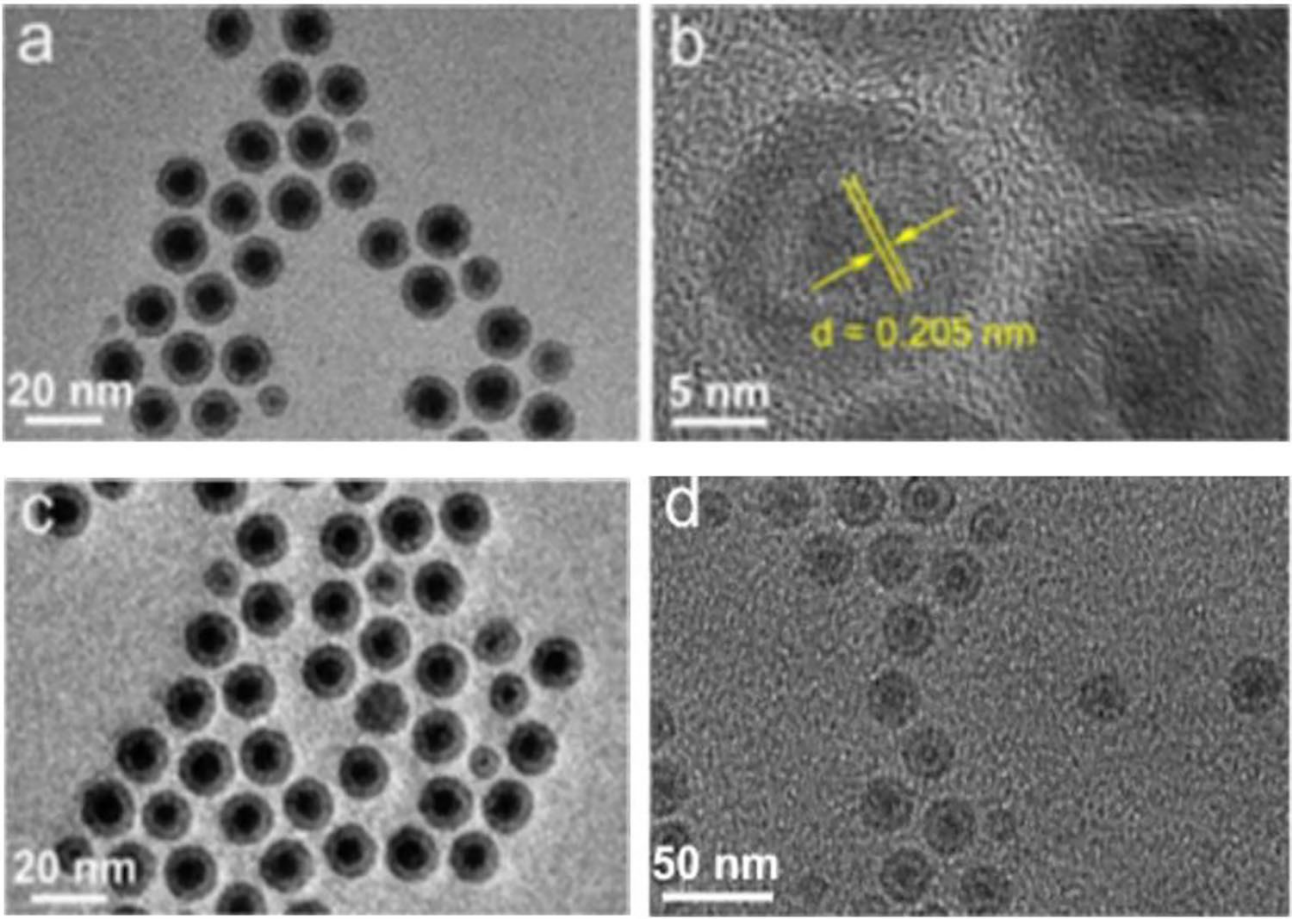
Microscopic images of Fe_5_C_2_-based nanoparticles. **a** Transmission electron microscopy (TEM) and **b** high-resolution TEM image of Fe_5_C_2_ nanoparticles, **c** TEM image of Fe_5_C_2_-GOD, and **d** Fe_5_C_2_-GOD@MnO_2_ nanocatalysts. Reproduced from [254] with permission from American Chemical Society (ACS)

A careful arrangement of particles forming aggregate nanoparticles can show enhanced sensitivity during detection when the nanoparticles are linked using unique, organic molecules or DNA [252]. Several nanoparticles have been prepared, including noble metals like Au. As molecular imaging detection depends on specific biomarkers' detection, the presence of nanomaterials bound.3 to specific ligands upon tissue cells can identify infected tissues like tumors [253]. Furthermore, nanomaterials with an ability to transport ions rather than electrons also artificially form suitable electrochemical detectors (Fig. 7).

## Conclusion

Cervical cancer is among the different types of cancers affecting women worldwide with significant morbidity and mortality. Trace elements though present in minute amount within the body, play a significant role in execution of biochemical pathways of cancer. The biological role of trace elements, particularly Cu, Zn, Se, Fe, As, Cd, and Mn, influence occurrence, incidence, proliferation, and mitigation of tumors. Fluctuations in the intracellular and extracellular levels of trace elements make them a viable option in diagnosis and therapy of cervical cancer. The incorporation of trace elements and minerals within the nanoparticulate systems provides an attractive strategy for the management of invasive cancers. Besides, supplementing trace elements during the occurrence of cervical cancer will effectively counteract tumor development. Trace elements have shown to exhibit great potential and hence will broaden the new therapeutic approaches for diagnosis and treatment of cervical cancer caused by both HPV and non-HPV induced cervical cancer.

## Abbreviations

HPV: Human Papillomavirus
LEEP: Loop electrosurgical procedure
Cu: Copper
Se: Selenium
Mn: Manganese
Fe: Iron
Zn: Zinc
Cd: Cadmium
As: Arsenic
ROS: Reactive oxygen species
DNA: Deoxyribonucleic acid
RNA: Ribonucleic acid
SOD: Superoxide dismutase
CIN: Cervical intraepithelial neoplasia
IARC: International Agency for Research on Cancer
HIV: Human immunodeficiency virus
PID: Pelvic inflammatory disease
SCC: Squamous Cell Carcinoma
SIL: Squamous intraepithelial lesion
LCR: Long control region
NCR: Non-coding region
MAPKs: Mitogen-stimulated pathway of protein kinase
AP-1: Activator protein-1
NFkB: Nuclear factor kappa B
TCT: Thin prep Cytological test
HC II: Hybrid Capture II
GPx: Glutathione peroxidase
Ru: Ruthenium
Cyt C: Cytochrome C
GOD: Glucose oxidase
CdS: Cadmium sulfide
BSA: Bovine serum albumin
QDs: Quantum dots
ZnO: Zinc oxide
ZnS: Zinc Sulfide
ZnSe: Zinc Selenide
ECL: Electrochemiluminescence
ROCK: Rho-associated kinase
Pdots: Polymer dots
NPs: Nanoparticles
